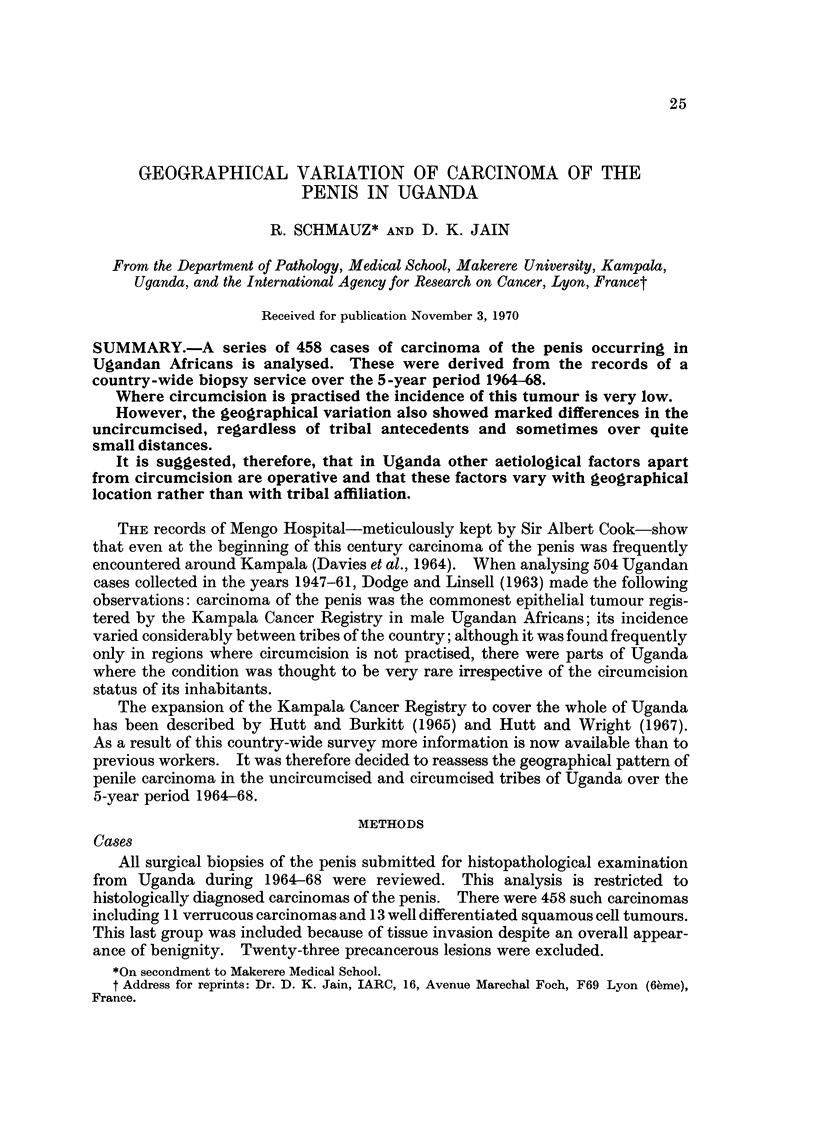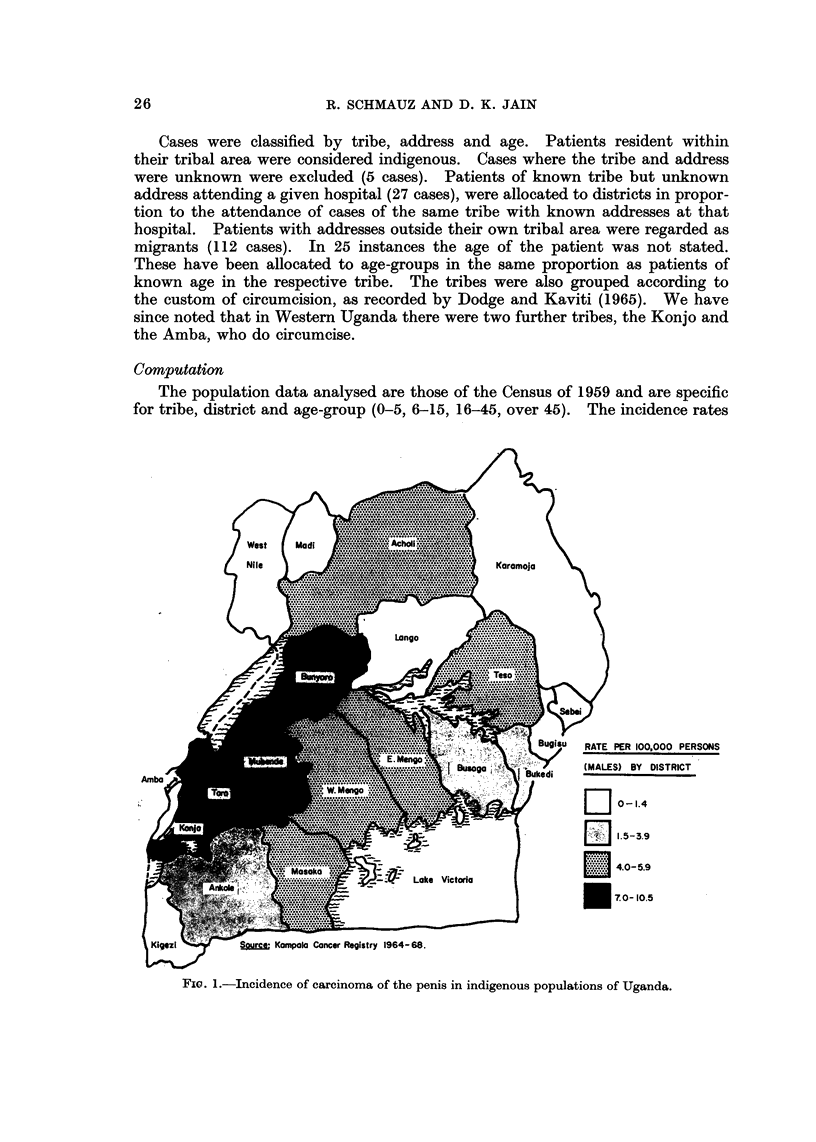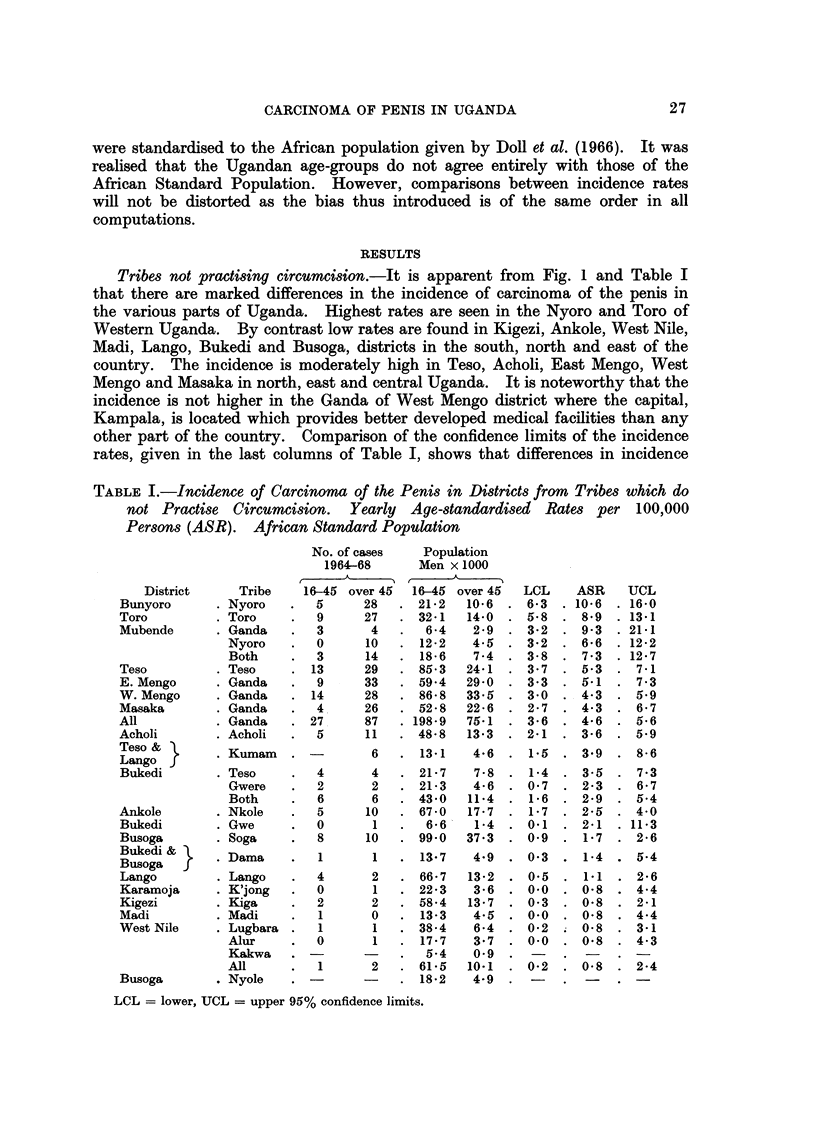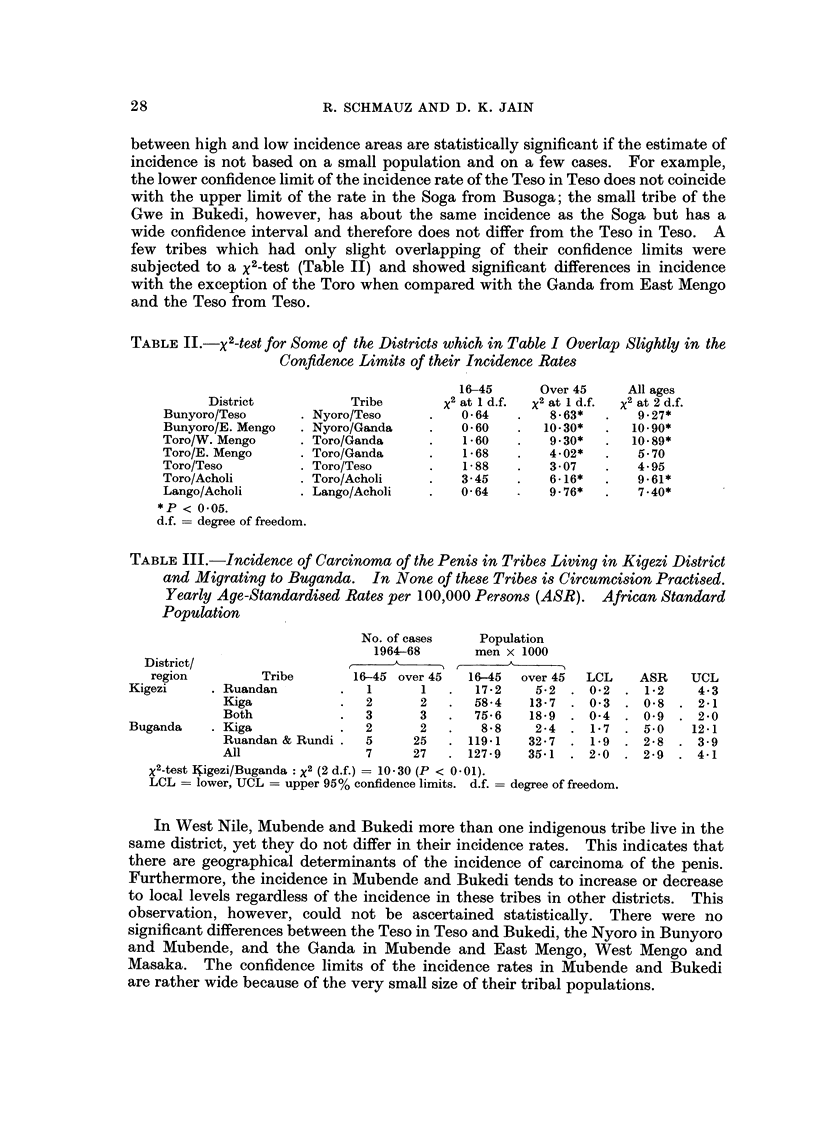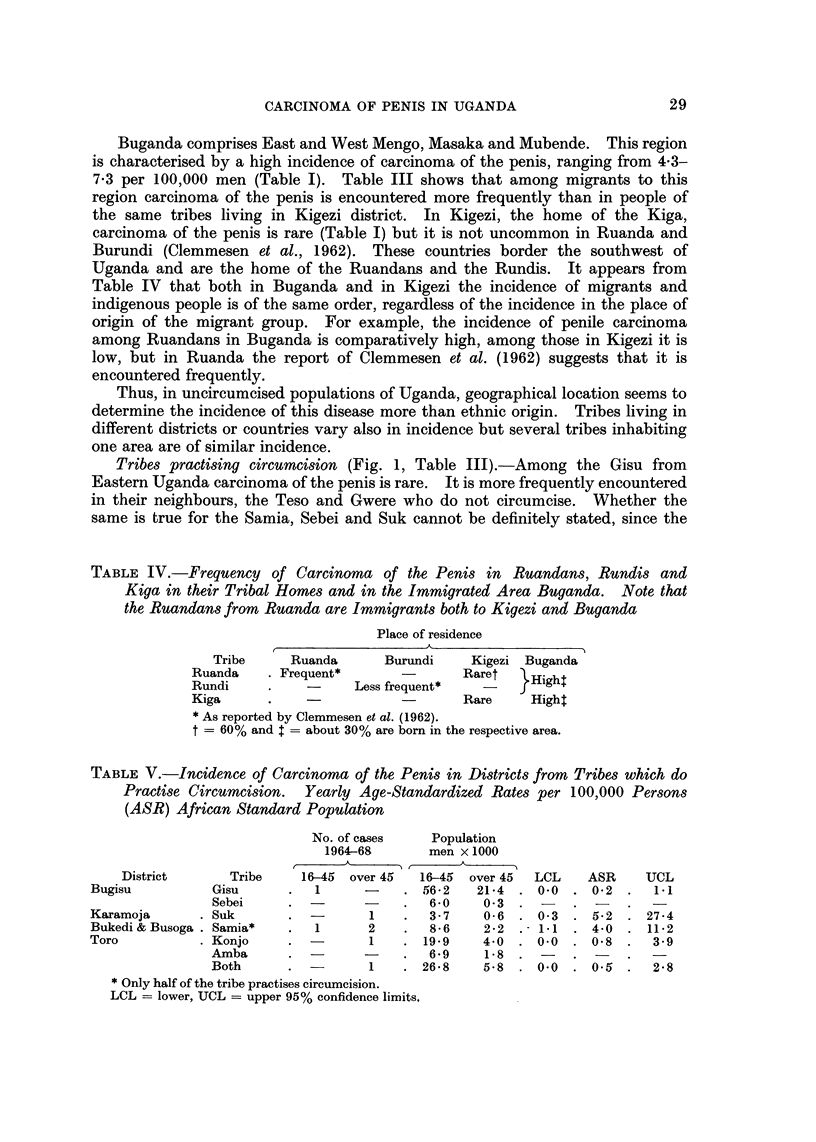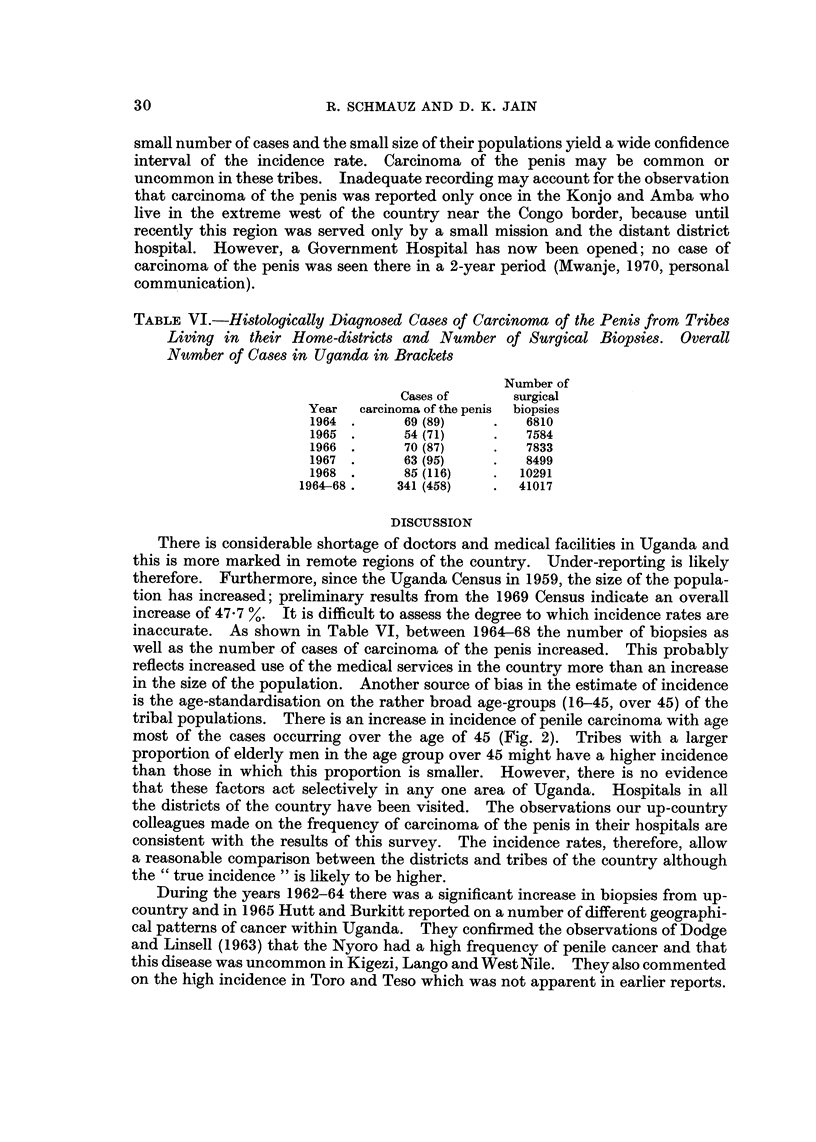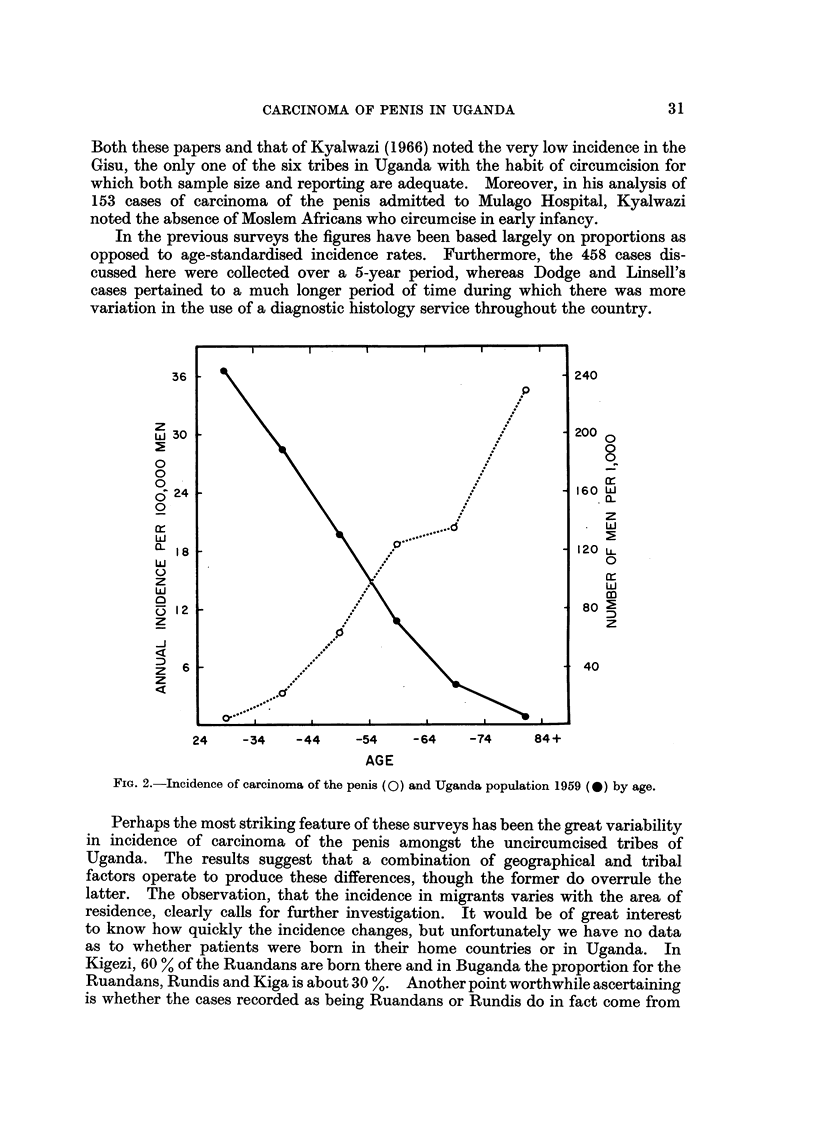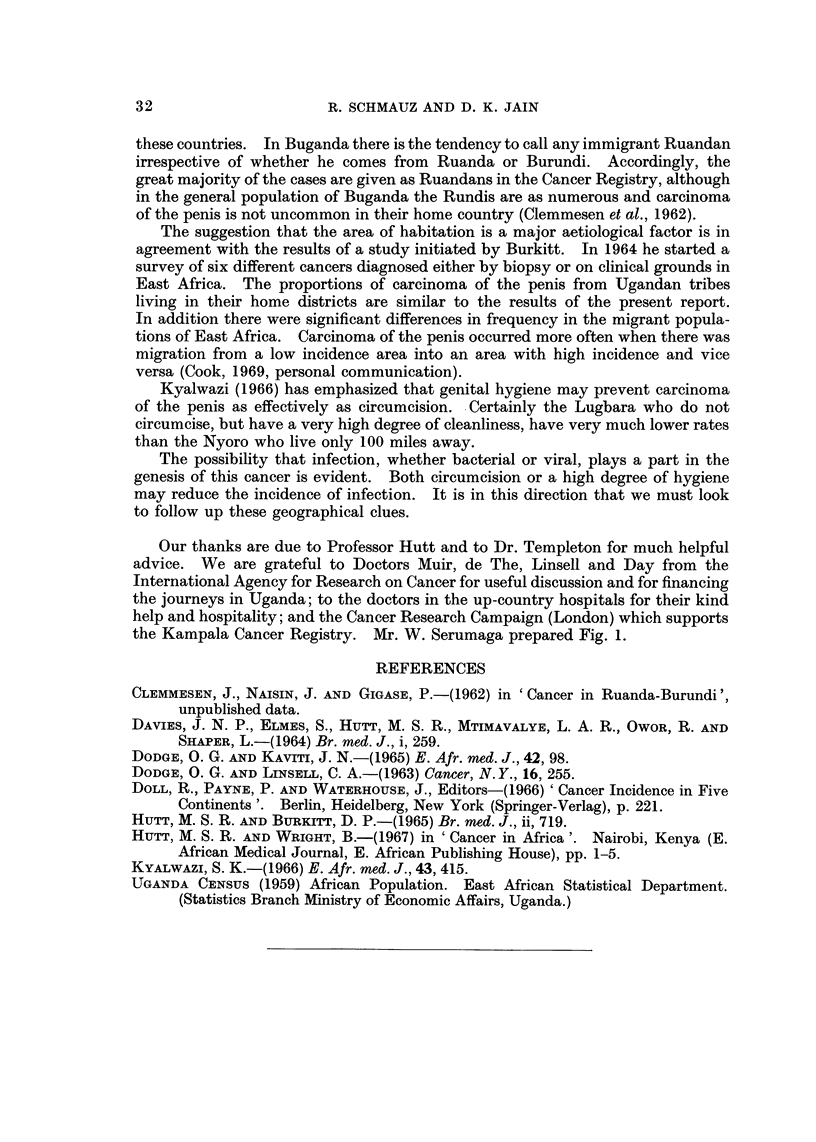# Geographical Variation of Carcinoma of the Penis in Uganda

**DOI:** 10.1038/bjc.1971.4

**Published:** 1971-03

**Authors:** R. Schmauz, D. K. Jain

## Abstract

A series of 458 cases of carcinoma of the penis occurring in Ugandan Africans is analysed. These were derived from the records of a country-wide biopsy service over the 5-year period 1964-68.

Where circumcision is practised the incidence of this tumour is very low.

However, the geographical variation also showed marked differences in the uncircumcised, regardless of tribal antecedents and sometimes over quite small distances.

It is suggested, therefore, that in Uganda other aetiological factors apart from circumcision are operative and that these factors vary with geographical location rather than with tribal affiliation.


### GEOGRAPHICAL VARIATION OF CARCINOMA OF THE PENIS IN UGANDA

R. SCHMAUZ* AND D. K. JAIN

*From the Department of Pathology, Medical School, Makerere University, Kampala, Uganda, and the International Agency for Research on Cancer, Lyon, France*†

Received for publication November 3, 1970

**SUMMARY.—A series of 458 cases of carcinoma of the penis occurring in Ugandan Africans is analysed. These were derived from the records of a country-wide biopsy service over the 5-year period 1964–68.**

**Where circumcision is practised the incidence of this tumour is very low.**

**However, the geographical variation also showed marked differences in the uncircumcised, regardless of tribal antecedents and sometimes over quite small distances.**

**It is suggested, therefore, that in Uganda other aetiological factors apart from circumcision are operative and that these factors vary with geographical location rather than with tribal affiliation.**

THE records of Mengo Hospital—meticulously kept by Sir Albert Cook—show that even at the beginning of this century carcinoma of the penis was frequently encountered around Kampala (Davies *et al.*, 1964). When analysing 504 Ugandan cases collected in the years 1947–61, Dodge and Linsell (1963) made the following observations: carcinoma of the penis was the commonest epithelial tumour registered by the Kampala Cancer Registry in male Ugandan Africans; its incidence varied considerably between tribes of the country; although it was found frequently only in regions where circumcision is not practised, there were parts of Uganda where the condition was thought to be very rare irrespective of the circumcision status of its inhabitants.

The expansion of the Kampala Cancer Registry to cover the whole of Uganda has been described by Hutt and Burkitt (1965) and Hutt and Wright (1967). As a result of this country-wide survey more information is now available than to previous workers. It was therefore decided to reassess the geographical pattern of penile carcinoma in the uncircumcised and circumcised tribes of Uganda over the 5-year period 1964–68.

### METHODS

### *Cases*

All surgical biopsies of the penis submitted for histopathological examination from Uganda during 1964–68 were reviewed. This analysis is restricted to histologically diagnosed carcinomas of the penis. There were 458 such carcinomas including 11 verrucous carcinomas and 13 well differentiated squamous cell tumours. This last group was included because of tissue invasion despite an overall appearance of benignity. Twenty-three precancerous lesions were excluded.

*On secondment to Makerere Medical School.

† Address for reprints: Dr. D. K. Jain, IARC, 16, Avenue Marechal Foch, F69 Lyon (6ème), France.

Cases were classified by tribe, address and age. Patients resident within their tribal area were considered indigenous. Cases where the tribe and address were unknown were excluded (5 cases). Patients of known tribe but unknown address attending a given hospital (27 cases), were allocated to districts in proportion to the attendance of cases of the same tribe with known addresses at that hospital. Patients with addresses outside their own tribal area were regarded as migrants (112 cases). In 25 instances the age of the patient was not stated. These have been allocated to age-groups in the same proportion as patients of known age in the respective tribe. The tribes were also grouped according to the custom of circumcision, as recorded by Dodge and Kaviti (1965). We have since noted that in Western Uganda there were two further tribes, the Konjo and the Amba, who do circumcise.

*Computation*

The population data analysed are those of the Census of 1959 and are specific for tribe, district and age-group (0–5, 6–15, 16–45, over 45). The incidence rates

FIG. 1.—Incidence of carcinoma of the penis in indigenous populations of Uganda.

were standardised to the African population given by Doll *et al.* (1966). It was realised that the Ugandan age-groups do not agree entirely with those of the African Standard Population. However, comparisons between incidence rates will not be distorted as the bias thus introduced is of the same order in all computations.

### RESULTS

*Tribes not practising circumcision.*—It is apparent from Fig. 1 and Table I that there are marked differences in the incidence of carcinoma of the penis in the various parts of Uganda. Highest rates are seen in the Nyoro and Toro of Western Uganda. By contrast low rates are found in Kigezi, Ankole, West Nile, Madi, Lango, Bukedi and Busoga, districts in the south, north and east of the country. The incidence is moderately high in Teso, Acholi, East Mengo, West Mengo and Masaka in north, east and central Uganda. It is noteworthy that the incidence is not higher in the Ganda of West Mengo district where the capital, Kampala, is located which provides better developed medical facilities than any other part of the country. Comparison of the confidence limits of the incidence rates, given in the last columns of Table I, shows that differences in incidence

TABLE I.—*Incidence of Carcinoma of the Penis in Districts from Tribes which do not Practise Circumcision. Yearly Age-standardised Rates per* 100,000 *Persons* (*ASR*). *African Standard Population*

| | | No. of cases 1964–68 | | Population Men × 1000 | | | | |
|---|---|---|---|---|---|---|---|---|
| District | Tribe | 16–45 | over 45 | 16–45 | over 45 | LCL | ASR | UCL |
| Bunyoro | Nyoro | 5 | 28 | 21·2 | 10·6 | 6·3 | 10·6 | 16·0 |
| Toro | Toro | 9 | 27 | 32·1 | 14·0 | 5·8 | 8·9 | 13·1 |
| Mubende | Ganda | 3 | 4 | 6·4 | 2·9 | 3·2 | 9·3 | 21·1 |
| | Nyoro | 0 | 10 | 12·2 | 4·5 | 3·2 | 6·6 | 12·2 |
| | Both | 3 | 14 | 18·6 | 7·4 | 3·8 | 7·3 | 12·7 |
| Teso | Teso | 13 | 29 | 85·3 | 24·1 | 3·7 | 5·3 | 7·1 |
| E. Mengo | Ganda | 9 | 33 | 59·4 | 29·0 | 3·3 | 5·1 | 7·3 |
| W. Mengo | Ganda | 14 | 28 | 86·8 | 33·5 | 3·0 | 4·3 | 5·9 |
| Masaka | Ganda | 4 | 26 | 52·8 | 22·6 | 2·7 | 4·3 | 6·7 |
| All | Ganda | 27 | 87 | 198·9 | 75·1 | 3·6 | 4·6 | 5·6 |
| Acholi | Acholi | 5 | 11 | 48·8 | 13·3 | 2·1 | 3·6 | 5·9 |
| Teso & Lango | Kumam | — | 6 | 13·1 | 4·6 | 1·5 | 3·9 | 8·6 |
| Bukedi | Teso | 4 | 4 | 21·7 | 7·8 | 1·4 | 3·5 | 7·3 |
| | Gwere | 2 | 2 | 21·3 | 4·6 | 0·7 | 2·3 | 6·7 |
| | Both | 6 | 6 | 43·0 | 11·4 | 1·6 | 2·9 | 5·4 |
| Ankole | Nkole | 5 | 10 | 67·0 | 17·7 | 1·7 | 2·5 | 4·0 |
| Bukedi | Gwe | 0 | 1 | 6·6 | 1·4 | 0·1 | 2·1 | 11·3 |
| Busoga | Soga | 8 | 10 | 99·0 | 37·3 | 0·9 | 1·7 | 2·6 |
| Bukedi & Busoga | Dama | 1 | 1 | 13·7 | 4·9 | 0·3 | 1·4 | 5·4 |
| Lango | Lango | 4 | 2 | 66·7 | 13·2 | 0·5 | 1·1 | 2·6 |
| Karamoja | K'jong | 0 | 1 | 22·3 | 3·6 | 0·0 | 0·8 | 4·4 |
| Kigezi | Kiga | 2 | 2 | 58·4 | 13·7 | 0·3 | 0·8 | 2·1 |
| Madi | Madi | 1 | 0 | 13·3 | 4·5 | 0·0 | 0·8 | 4·4 |
| West Nile | Lugbara | 1 | 1 | 38·4 | 6·4 | 0·2 | 0·8 | 3·1 |
| | Alur | 0 | 1 | 17·7 | 3·7 | 0·0 | 0·8 | 4·3 |
| | Kakwa | — | — | 5·4 | 0·9 | — | — | — |
| | All | 1 | 2 | 61·5 | 10·1 | 0·2 | 0·8 | 2·4 |
| Busoga | Nyole | — | — | 18·2 | 4·9 | — | — | — |

LCL = lower, UCL = upper 95% confidence limits.

between high and low incidence areas are statistically significant if the estimate of incidence is not based on a small population and on a few cases. For example, the lower confidence limit of the incidence rate of the Teso in Teso does not coincide with the upper limit of the rate in the Soga from Busoga; the small tribe of the Gwe in Bukedi, however, has about the same incidence as the Soga but has a wide confidence interval and therefore does not differ from the Teso in Teso. A few tribes which had only slight overlapping of their confidence limits were subjected to a $\chi^2$-test (Table II) and showed significant differences in incidence with the exception of the Toro when compared with the Ganda from East Mengo and the Teso from Teso.

TABLE II.—*$\chi^2$-test for Some of the Districts which in Table I Overlap Slightly in the Confidence Limits of their Incidence Rates*

| District | Tribe | 16–45 $\chi^2$ at 1 d.f. | Over 45 $\chi^2$ at 1 d.f. | All ages $\chi^2$ at 2 d.f. |
|---|---|---|---|---|
| Bunyoro/Teso | Nyoro/Teso | 0·64 | 8·63* | 9·27* |
| Bunyoro/E. Mengo | Nyoro/Ganda | 0·60 | 10·30* | 10·90* |
| Toro/W. Mengo | Toro/Ganda | 1·60 | 9·30* | 10·89* |
| Toro/E. Mengo | Toro/Ganda | 1·68 | 4·02* | 5·70 |
| Toro/Teso | Toro/Teso | 1·88 | 3·07 | 4·95 |
| Toro/Acholi | Toro/Acholi | 3·45 | 6·16* | 9·61* |
| Lango/Acholi | Lango/Acholi | 0·64 | 9·76* | 7·40* |

*$P < 0·05$.
d.f. = degree of freedom.

TABLE III.—*Incidence of Carcinoma of the Penis in Tribes Living in Kigezi District and Migrating to Buganda. In None of these Tribes is Circumcision Practised. Yearly Age-Standardised Rates per* 100,000 *Persons* (*ASR*). *African Standard Population*

| District/ region | Tribe | No. of cases 1964–68 16–45 | over 45 | Population men × 1000 16–45 | over 45 | LCL | ASR | UCL |
|---|---|---|---|---|---|---|---|---|
| Kigezi | Ruandan | 1 | 1 | 17·2 | 5·2 | 0·2 | 1·2 | 4·3 |
| | Kiga | 2 | 2 | 58·4 | 13·7 | 0·3 | 0·8 | 2·1 |
| | Both | 3 | 3 | 75·6 | 18·9 | 0·4 | 0·9 | 2·0 |
| Buganda | Kiga | 2 | 2 | 8·8 | 2·4 | 1·7 | 5·0 | 12·1 |
| | Ruandan & Rundi | 5 | 25 | 119·1 | 32·7 | 1·9 | 2·8 | 3·9 |
| | All | 7 | 27 | 127·9 | 35·1 | 2·0 | 2·9 | 4·1 |

$\chi^2$-test Kigezi/Buganda : $\chi^2$ (2 d.f.) = 10·30 ($P < 0·01$).
LCL = lower, UCL = upper 95% confidence limits. d.f. = degree of freedom.

In West Nile, Mubende and Bukedi more than one indigenous tribe live in the same district, yet they do not differ in their incidence rates. This indicates that there are geographical determinants of the incidence of carcinoma of the penis. Furthermore, the incidence in Mubende and Bukedi tends to increase or decrease to local levels regardless of the incidence in these tribes in other districts. This observation, however, could not be ascertained statistically. There were no significant differences between the Teso in Teso and Bukedi, the Nyoro in Bunyoro and Mubende, and the Ganda in Mubende and East Mengo, West Mengo and Masaka. The confidence limits of the incidence rates in Mubende and Bukedi are rather wide because of the very small size of their tribal populations.

Buganda comprises East and West Mengo, Masaka and Mubende. This region is characterised by a high incidence of carcinoma of the penis, ranging from 4·3–7·3 per 100,000 men (Table I). Table III shows that among migrants to this region carcinoma of the penis is encountered more frequently than in people of the same tribes living in Kigezi district. In Kigezi, the home of the Kiga, carcinoma of the penis is rare (Table I) but it is not uncommon in Ruanda and Burundi (Clemmesen *et al.*, 1962). These countries border the southwest of Uganda and are the home of the Ruandans and the Rundis. It appears from Table IV that both in Buganda and in Kigezi the incidence of migrants and indigenous people is of the same order, regardless of the incidence in the place of origin of the migrant group. For example, the incidence of penile carcinoma among Ruandans in Buganda is comparatively high, among those in Kigezi it is low, but in Ruanda the report of Clemmesen *et al.* (1962) suggests that it is encountered frequently.

Thus, in uncircumcised populations of Uganda, geographical location seems to determine the incidence of this disease more than ethnic origin. Tribes living in different districts or countries vary also in incidence but several tribes inhabiting one area are of similar incidence.

*Tribes practising circumcision* (Fig. 1, Table III).—Among the Gisu from Eastern Uganda carcinoma of the penis is rare. It is more frequently encountered in their neighbours, the Teso and Gwere who do not circumcise. Whether the same is true for the Samia, Sebei and Suk cannot be definitely stated, since the

TABLE IV.—*Frequency of Carcinoma of the Penis in Ruandans, Rundis and Kiga in their Tribal Homes and in the Immigrated Area Buganda. Note that the Ruandans from Ruanda are Immigrants both to Kigezi and Buganda*

| | Place of residence | | | |
|---|---|---|---|---|
| Tribe | Ruanda | Burundi | Kigezi | Buganda |
| Ruanda | Frequent* | — | Rare† | High‡ (Ruanda and Rundi) |
| Rundi | — | Less frequent* | — | |
| Kiga | — | — | Rare | High‡ |

* As reported by Clemmesen *et al.* (1962).
† = 60% and ‡ = about 30% are born in the respective area.

TABLE V.—*Incidence of Carcinoma of the Penis in Districts from Tribes which do Practise Circumcision. Yearly Age-Standardized Rates per* 100,000 *Persons* (*ASR*) *African Standard Population*

| | | No. of cases 1964–68 | | Population men × 1000 | | | | |
|---|---|---|---|---|---|---|---|---|
| District | Tribe | 16–45 | over 45 | 16–45 | over 45 | LCL | ASR | UCL |
| Bugisu | Gisu | 1 | — | 56·2 | 21·4 | 0·0 | 0·2 | 1·1 |
| | Sebei | — | — | 6·0 | 0·3 | — | — | — |
| Karamoja | Suk | — | 1 | 3·7 | 0·6 | 0·3 | 5·2 | 27·4 |
| Bukedi & Busoga | Samia* | 1 | 2 | 8·6 | 2·2 | 1·1 | 4·0 | 11·2 |
| Toro | Konjo | — | 1 | 19·9 | 4·0 | 0·0 | 0·8 | 3·9 |
| | Amba | — | — | 6·9 | 1·8 | — | — | — |
| | Both | — | 1 | 26·8 | 5·8 | 0·0 | 0·5 | 2·8 |

* Only half of the tribe practises circumcision.
LCL = lower, UCL = upper 95% confidence limits.

small number of cases and the small size of their populations yield a wide confidence interval of the incidence rate. Carcinoma of the penis may be common or uncommon in these tribes. Inadequate recording may account for the observation that carcinoma of the penis was reported only once in the Konjo and Amba who live in the extreme west of the country near the Congo border, because until recently this region was served only by a small mission and the distant district hospital. However, a Government Hospital has now been opened; no case of carcinoma of the penis was seen there in a 2-year period (Mwanje, 1970, personal communication).

TABLE VI.—*Histologically Diagnosed Cases of Carcinoma of the Penis from Tribes Living in their Home-districts and Number of Surgical Biopsies. Overall Number of Cases in Uganda in Brackets*

| Year | Cases of carcinoma of the penis | Number of surgical biopsies |
|---|---|---|
| 1964 | 69 (89) | 6810 |
| 1965 | 54 (71) | 7584 |
| 1966 | 70 (87) | 7833 |
| 1967 | 63 (95) | 8499 |
| 1968 | 85 (116) | 10291 |
| 1964–68 | 341 (458) | 41017 |

### DISCUSSION

There is considerable shortage of doctors and medical facilities in Uganda and this is more marked in remote regions of the country. Under-reporting is likely therefore. Furthermore, since the Uganda Census in 1959, the size of the population has increased; preliminary results from the 1969 Census indicate an overall increase of 47·7 %. It is difficult to assess the degree to which incidence rates are inaccurate. As shown in Table VI, between 1964–68 the number of biopsies as well as the number of cases of carcinoma of the penis increased. This probably reflects increased use of the medical services in the country more than an increase in the size of the population. Another source of bias in the estimate of incidence is the age-standardisation on the rather broad age-groups (16–45, over 45) of the tribal populations. There is an increase in incidence of penile carcinoma with age most of the cases occurring over the age of 45 (Fig. 2). Tribes with a larger proportion of elderly men in the age group over 45 might have a higher incidence than those in which this proportion is smaller. However, there is no evidence that these factors act selectively in any one area of Uganda. Hospitals in all the districts of the country have been visited. The observations our up-country colleagues made on the frequency of carcinoma of the penis in their hospitals are consistent with the results of this survey. The incidence rates, therefore, allow a reasonable comparison between the districts and tribes of the country although the " true incidence " is likely to be higher.

During the years 1962–64 there was a significant increase in biopsies from up-country and in 1965 Hutt and Burkitt reported on a number of different geographical patterns of cancer within Uganda. They confirmed the observations of Dodge and Linsell (1963) that the Nyoro had a high frequency of penile cancer and that this disease was uncommon in Kigezi, Lango and West Nile. They also commented on the high incidence in Toro and Teso which was not apparent in earlier reports.

Both these papers and that of Kyalwazi (1966) noted the very low incidence in the Gisu, the only one of the six tribes in Uganda with the habit of circumcision for which both sample size and reporting are adequate. Moreover, in his analysis of 153 cases of carcinoma of the penis admitted to Mulago Hospital, Kyalwazi noted the absence of Moslem Africans who circumcise in early infancy.

In the previous surveys the figures have been based largely on proportions as opposed to age-standardised incidence rates. Furthermore, the 458 cases discussed here were collected over a 5-year period, whereas Dodge and Linsell's cases pertained to a much longer period of time during which there was more variation in the use of a diagnostic histology service throughout the country.

FIG. 2.—Incidence of carcinoma of the penis (○) and Uganda population 1959 (●) by age.

Perhaps the most striking feature of these surveys has been the great variability in incidence of carcinoma of the penis amongst the uncircumcised tribes of Uganda. The results suggest that a combination of geographical and tribal factors operate to produce these differences, though the former do overrule the latter. The observation, that the incidence in migrants varies with the area of residence, clearly calls for further investigation. It would be of great interest to know how quickly the incidence changes, but unfortunately we have no data as to whether patients were born in their home countries or in Uganda. In Kigezi, 60 % of the Ruandans are born there and in Buganda the proportion for the Ruandans, Rundis and Kiga is about 30 %. Another point worthwhile ascertaining is whether the cases recorded as being Ruandans or Rundis do in fact come from these countries. In Buganda there is the tendency to call any immigrant Ruandan irrespective of whether he comes from Ruanda or Burundi. Accordingly, the great majority of the cases are given as Ruandans in the Cancer Registry, although in the general population of Buganda the Rundis are as numerous and carcinoma of the penis is not uncommon in their home country (Clemmesen *et al.*, 1962).

The suggestion that the area of habitation is a major aetiological factor is in agreement with the results of a study initiated by Burkitt. In 1964 he started a survey of six different cancers diagnosed either by biopsy or on clinical grounds in East Africa. The proportions of carcinoma of the penis from Ugandan tribes living in their home districts are similar to the results of the present report. In addition there were significant differences in frequency in the migrant populations of East Africa. Carcinoma of the penis occurred more often when there was migration from a low incidence area into an area with high incidence and vice versa (Cook, 1969, personal communication).

Kyalwazi (1966) has emphasized that genital hygiene may prevent carcinoma of the penis as effectively as circumcision. Certainly the Lugbara who do not circumcise, but have a very high degree of cleanliness, have very much lower rates than the Nyoro who live only 100 miles away.

The possibility that infection, whether bacterial or viral, plays a part in the genesis of this cancer is evident. Both circumcision or a high degree of hygiene may reduce the incidence of infection. It is in this direction that we must look to follow up these geographical clues.

Our thanks are due to Professor Hutt and to Dr. Templeton for much helpful advice. We are grateful to Doctors Muir, de The, Linsell and Day from the International Agency for Research on Cancer for useful discussion and for financing the journeys in Uganda; to the doctors in the up-country hospitals for their kind help and hospitality; and the Cancer Research Campaign (London) which supports the Kampala Cancer Registry. Mr. W. Serumaga prepared Fig. 1.

### REFERENCES

Clemmesen, J., Naisin, J. and Gigase, P.—(1962) in ' Cancer in Ruanda-Burundi ', unpublished data.

Davies, J. N. P., Elmes, S., Hutt, M. S. R., Mtimavalye, L. A. R., Owor, R. and Shaper, L.—(1964) *Br. med. J.*, i, 259.

Dodge, O. G. and Kaviti, J. N.—(1965) *E. Afr. med. J.*, **42**, 98.

Dodge, O. G. and Linsell, C. A.—(1963) *Cancer, N.Y.*, **16**, 255.

Doll, R., Payne, P. and Waterhouse, J., Editors—(1966) ' Cancer Incidence in Five Continents '. Berlin, Heidelberg, New York (Springer-Verlag), p. 221.

Hutt, M. S. R. and Burkitt, D. P.—(1965) *Br. med. J.*, ii, 719.

Hutt, M. S. R. and Wright, B.—(1967) in ' Cancer in Africa '. Nairobi, Kenya (E. African Medical Journal, E. African Publishing House), pp. 1–5.

Kyalwazi, S. K.—(1966) *E. Afr. med. J.*, **43**, 415.

Uganda Census (1959) African Population. East African Statistical Department. (Statistics Branch Ministry of Economic Affairs, Uganda.)